# Pinitol Improves Diabetic Foot Ulcers in Streptozotocin-Induced Diabetes Rats Through Upregulation of Nrf2/HO-1 Signaling

**DOI:** 10.3390/antiox14010015

**Published:** 2024-12-26

**Authors:** Jinsick Kim, Min Young Go, Chae Young Jeon, Jung Un Shin, Mujun Kim, Hye Won Lim, Dong Wook Shin

**Affiliations:** Research Institute for Biomedical and Health Science, Konkuk University, Chungju 27478, Republic of Korea; jindoli477@kku.ac.kr (J.K.); rhalsdud1011@kku.ac.kr (M.Y.G.); young4mam@kku.ac.kr (C.Y.J.); ibd5252@kku.ac.kr (J.U.S.); besy100@kku.ac.kr (M.K.); hyewon0225@kku.ac.kr (H.W.L.)

**Keywords:** pinitol, diabetic foot ulcers, streptozotocin, diabetic rat, Nrf2

## Abstract

Diabetic foot ulcers represent a severe complication of diabetes, often resulting in amputation and high mortality rates. Currently, there are no treatments for diabetic foot ulcers other than antibiotics and dressings. In this study, we evaluated the wound-healing effects of an antidiabetic agent pinitol in lipopolysaccharide (LPS)-damaged human dermal fibroblasts (HDFs) and streptozotocin (STZ)-induced diabetic rat models with a foot wound. Our findings indicated that pinitol enhanced cell migration, proliferation, and wound healing by activating Nrf2, thereby mitigating oxidative stress and inflammatory responses at the wound site. Additionally, pinitol restored mitochondrial energy metabolism, decreased matrix metalloproteinase (MMP) activity, and increased collagen deposition. Furthermore, pinitol facilitated angiogenesis, contributing to improved wound healing. Taken together, these findings suggest that pinitol could be a promising therapeutic agent for the treatment of diabetic foot ulcers.

## 1. Introduction

Diabetes mellitus (DM) is a chronic disorder resulting from impaired glucose metabolism. The accumulation of glucose in the blood of diabetic patients leads to chronic damage to organs and tissues, resulting in severe complications such as retinopathy, nephropathy, neuropathy, and non-healing skin ulcers, with a prevalence of over 400 million individuals worldwide [1,2]. Among these, 15–25% develop severe foot ulcers. The 5-year mortality rate for patients undergoing lower limb amputation due to foot ulcers is 68%, and 60% of patients who recover from diabetic foot ulcers relapse within three years [3,4,5].

In diabetic foot ulcers, prolonged inflammation has a significant impact on chronic wound formation. When microbial colonization occurs in the ulcer site, it becomes difficult to control, ultimately leading to severe infections [6]. Such infections are particularly challenging for diabetic patients, hindering wound healing and recovery. Despite novel treatment modalities such as growth factor therapies, nano-formulations, microRNA-based treatments, and skin grafting approaches aimed at enhancing complex wound-healing mechanisms and addressing the need for long-term care, effective clinical management and evaluation of chronic wounds remain a challenge for healthcare systems. This highlights the persistent necessity for personalized and efficient therapeutic strategies that can adapt to the complex nature of chronic ulcers, tailored to their pathophysiological state [7].

Diabetic foot ulcers may arise from various factors, such as repeated infections, impaired angiogenesis, excessive ROS production, and induced inflammation [7]. In diabetic foot ulcers, the infection-induced hostile environment damages fibroblasts and vascular progenitor cells, leading to cell death, which impairs fundamental processes such as cell migration and proliferation that are essential for wound healing [8,9]. The early stage of responding to invading pathogens and skin injury involves initiating cell signaling pathways, which starts with increased ROS production [10]. Excessive ROS production in damaged and inflamed tissue assists in Nrf2 synthesis, which contributes to wound healing [11]. Nrf2 is a crucial signaling pathway that protects from environmental stressors, such as ROS, electrophilic, and protein-toxic stress [12]. Additionally, Nrf2 signaling helps inhibit pro-inflammatory cytokines, suppressing inflammation, and regulates basic cellular processes such as apoptosis, autophagy, angiogenesis, proliferation, and migration, thereby contributing to the cellular response to inflammation [11,13,14].

Pinitol, a methyl ether of D-chiro-inositol (DCI), is found abundantly in soy and other leguminous foods and is known for its potent antioxidant, anticancer, anti-inflammatory, and wound-healing properties. Notably, pinitol is structurally like insulin and has been demonstrated to reduce blood glucose levels in insulin-deficient rats, leading to its classification as an antidiabetic agent. Furthermore, pinitol has been found to prevent endothelial dysfunction induced by diabetes, attributed to its antioxidant capabilities [15,16,17]. Our previous study also observed that pinitol enhances wound healing in ultraviolet-induced skin damage [18].

Despite its potent antioxidants, anti-inflammatory, antidiabetic, and wound-healing properties, pinitol has not yet been studied for its potential in treating diabetic foot ulcers. Therefore, this study aimed to explore novel therapeutic targets and potential mechanisms of pinitol for treating diabetic foot ulcers.

## 2. Materials and Methods

### 2.1. Cell Culture

Human Dermal Fibroblast (HDFs) were obtained from PromoCell (Heidelberg, Germany) and cultured in 10% (*v*/*v*) fetal bovine serum (FBS)-high-glucose DMEM (Welgene, Gyeongsangbuk-do, Republic of Korea) in a 5% CO_2_ incubator at 37 °C.

### 2.2. Cell Viability Assay

The cytotoxicity of the pinitol, metformin, and LPS (Sigma-Aldrich, St. Louis, MO, USA) was evaluated using HDFs. They were seeded in a cell culture plate with 96wells. Cells were incubated with 2.5~100 μM pinitol, 25~1000 μM metformin, or 1 μg/mL LPS for 24 and 48 h. The cell viability (*n* = 6) was assessed by the EZ-Cytox cell viability assay kit (DoGenBio, Seoul, Republic of Korea) and a microplate reader (BioTek Multi-Mode Microplate Reader, Winooski, VT, USA) at 450 and 600 nm. The cell viability was calculated based on the UV absorbance values of the samples compared with those of the control.

### 2.3. Cell Proliferation Assay

Cell proliferation was measured with the Click-iT EdU Alexa Fluor 488 Imaging Kit (Thermo Fisher Scientific, Waltham, MA, USA). HDFs were cultured in a confocal dish. Cells were incubated with 1 μg/mL LPS, 1 mM metformin, and 100 μM pinitol for 12–48 h. Cells were incubated for 24 h, with half of the media replaced with fresh media containing 20 μM EdU. The cells were then fixed with 4% paraformaldehyde (Thermo Fisher Scientific, Waltham, MA, USA) for 15 min and permeabilized with DPBS (Welgene, Gyeongsangbuk-do, Republic of Korea) with 0.5% Triton X-100 (Sigma-Aldrich, St. Louis, MO, USA) for 20 min. Then, each well was treated with a 500 µL Click-iT Plus reaction cocktail and incubated for 30 min. After washing three times with 3% BSA in DPBS, cells were incubated with 10 µg/mL Hoechst 33342 (Thermo Fisher Scientific , Waltham, MA, USA) for 15 min. After washing out the Hoechst 33342, cells were washed with DPBS three times. Images were captured using a Nikon Eclipse Ti2 fluorescence live-cell imaging microscope (Nikon, Tokyo, Japan).

### 2.4. Wound Healing Assay

Wound healing was assessed using a scratch assay. HDFs were seeded in a 6-well plate. When the cell density reached around 90%, a narrow wound was carefully created along the center of each well using a 200 μL pipette tip, and this moment was set as 0 h. Cells were washed out with DPBS and were added with each 1 μg/mL LPS, 1 mM metformin, and 100 μM pinitol and incubated for 24 h. To compare images 0 h and 24 h, the closed distance was quantitatively evaluated using ImageJ software version 2.9.0 (National Institutes of Health, MD, USA).

### 2.5. Measurement of ROS

The fluorescent marker 2′,7′-dichlorodihydrofluorescein diacetate (DCF-DA, Sigma-Aldrich, St. Louis, MO, USA) was used to confirm the generation of ROS from the LPS. HDFs were cultured in a confocal dish and incubated for 24 h. Cells were incubated with 1 μg/mL LPS, 1 mM metformin, and 100 μM pinitol for 24 h. The medium was removed, and cells were further incubated with DCF-DA (10 μM) in the dark. Fluorescence images were captured using a Nikon Eclipse Ti2 fluorescence live-cell imaging microscope through a FITC filter (Nikon, Tokyo, Japan).

### 2.6. Measurement of Mitochondrial Membrane Potential (JC-1)

The mitochondrial membrane potential was assessed by a JC-1 assay kit (Abcam, Cambridge, UK). HDFs were cultured in a confocal dish and incubated for 24 h. Cells were treated with 1 μg/mL LPS, 1 mM metformin, and 100 μM pinitol for 24 h. The medium was removed, and cells were further incubated with JC-1 (1 μM) at 37 °C for 10 min. Fluorescence images were captured using a Nikon Eclipse Ti2 fluorescence live-cell imaging microscope through Cy3/TRITC and FITC filters (Nikon, Tokyo, Japan).

### 2.7. Measurement of Mitochondrial ATP Content

Relative mitochondrial ATP content was measured using the Cell Meter™ Live Cell ATP Assay Kit (AAT Bioquest, Sunnyvale, CA, USA). HDFs were cultured in a confocal dish and incubated for 24 h. Cells were incubated with 1 μg/mL LPS, 1 mM metformin, and 100 μM pinitol for 24 h. The medium was removed, and cells were further incubated with ATP Red™ for 15 min and then incubated with MitoLite™ Green FM (AAT Bioquest, Sunnyvale, CA, USA) (200 nM) for another 30 min. Fluorescence images were captured using a Nikon Eclipse Ti2 fluorescence live-cell imaging microscope through Cy3/TRITC and FITC filters.

### 2.8. Nuclear and Cytoplasmic Protein Extraction

Nuclear and cytoplasmic protein extraction using the Cell Fractionation Kit. HDFs were cultured in a culture dish and incubated for 24 h. Cells were incubated with 1 μg/mL LPS, 1 mM metformin, and 100 μM pinitol for 24 h, followed by fractionation by Cell Fractionation Kit Standard (Abcam, Cambridge, UK). The protein concentration was evaluated using a BCA assay kit (Thermo Fisher Scientific, Waltham, MA, USA).

### 2.9. Western Blot Analysis

After sample treatment, cells were washed with cold DPBS and lysed on ice for 20 min using a cold RIPA lysis buffer (Thermo Fisher Scientific, Waltham, MA, USA). The lysates were collected and quantified using a BCA assay kit. After electrophoresis, the proteins were transferred to a polyvinylidene fluoride membrane. Then, the membranes were incubated with 5% milk at room temperature for 2 h. The blocked membranes were incubated overnight at 4 °C with specific antibodies p-p38, p38, p-ERK, ERK, p-JNK, JNK, p-NF-κB, NF-κB, Iκα, HO-1, Keap1, β-actin, and Lamin B (Cell Signaling Technology, Danvers, MA, USA), p-Iκα, p-Nrf2 (Invitrogen, Carlsbad, CA, USA), and Nrf2 (Novus Biologicals, Centennial, CO, USA), followed by incubation with HRP-conjugated secondary antibody for 2 h. The target proteins were visualized using an ECL reagent kit, and images were acquired with the Invitrogen iBright 1500 Imaging System (Thermo Fisher Scientific, Waltham, MA, USA) and were finally analyzed by ImageJ.

### 2.10. Immunofluorescence Analysis

HDFs were seeded in a confocal dish at a 4.0 × 10^4^ cells/well concentration and incubated for 24 h. Then, the cells were incubated with 1 μg/mL LPS, 1 mM metformin, and 100 μM pinitol for 24 h. Briefly, the cells were fixed with 4% paraformaldehyde for 15 min and permeabilized with 0.1% Triton X-100 at room temperature for 15 min. After blocking with 3% BSA for 1 h, the cells were incubated with p-Nrf2, or NF-κB at 4 °C for 12 h and incubated with HRP-conjugated secondary antibody at 37 °C for 1 h. Finally, the nuclei were stained with DAPI for 15 min. The fluorescence was detected with a Nikon Eclipse Ti2 fluorescence live-cell imaging microscope.

### 2.11. Real-Time Quantitative Reverse Transcriptase-Polymerase Chain Reaction 

HDFs were plated in a 6-well dish at 14.0 × 10^4^ cells/well. The cells were cultured for 24 h. Subsequently, the cells were treated with 1 μg/mL LPS, 1 mM metformin, and 100 μM pinitol for 24 h. Cells were washed twice with DPBS before being used for RNA extraction. RNA was isolated using TRIzol reagent (Thermo Fisher Scientific, Waltham, MA, USA), and 2 μg of total RNA was converted into cDNA using the RevertAid First Strand cDNA Synthesis Kit (Thermo Fisher Scientific, Waltham, MA, USA). The assays were performed utilizing TaqMan Universal Master Mix II, with UNG, for real-time quantitative reverse transcriptase-polymerase chain reaction (qRT-PCR). The reaction mixture consisted of DEPC water (6 μL), TaqMan Universal Master Fast Mix II (10 μL), cDNA (3 μL), and each primer (1 μL).

### 2.12. Animal Models of Diabetes

Male Sprague-Dawley (SD), Specific Pathogen-Free (SPF) rats (5 weeks old, 180–200 g, 48 animals) were purchased from Orient Bio (Seungnam, Republic of Korea). All rats were acclimatized for one week under standard laboratory conditions before the start of the experiment. They were randomly assigned to four groups: control group (*n* = 12), streptozotocin (STZ) group (*n* = 12), STZ + metformin group (*n* = 12), and STZ + pinitol group (*n* = 12). During the acclimatization period, rats had ad libitum access to both food and water while being housed in an animal facility. All animal experiments were conducted following the standard guidelines of the Institutional Animal Care and Use Committee (IACUC) at Konkuk University (reference number: KU24112). In this study, to investigate diabetic wound healing, STZ (purity ≥ 98%, Sigma-Aldrich, St. Louis, MO, USA) (65 mg/mL) was administered intravenously to 36 rats [19], while the control group received an equivalent volume of saline. Blood glucose levels were assessed 48 h after injection with STZ, and rats with blood glucose levels above 300 mg/dL were considered diabetic and selected for further experimentation.

### 2.13. Wound-Healing Model

In the diabetic foot wound healing model, control and diabetic rats were anesthetized with an intraperitoneal injection of 2,2,2-tribromoethanol (Sigma-Aldrich, St. Louis, MO, USA) at 240 mg/kg. A wound was created on the hind paw of each anesthetized rat using a 6 × 6 mm punch, penetrating to the dermal layer. Photographs of the wound area were taken, and wound size was measured every two days using calipers. The percentage of wound size reduction (%) was calculated as follows: Wound size reduction (%) = (At − A0)/A0 × 100, where A0 represents the wound area on the first day of wound creation, and At represents the wound area on the specified day. Metformin and pinitol treatments were applied twice daily until day 8. The treatment cream was prepared and applied topically to the wound area at a dose of 50 μL per application. The cream was formulated as follows for a 10 mL volume: pinitol 500 mg was dissolved in ddH_2_O 7 mL, followed by adding 1 mL 1,3-butanediol, 2 mL ethanol (Sigma-Aldrich, St. Louis, MO, USA), and carbomer 980 (WhatSoap, Gyeonggi-do, Republic of Korea) (0.1 mg). On day 8 post-wounding, the rats were sacrificed, and skin samples were collected for further analysis.

### 2.14. Measurement of MDA, and GSH Levels

The wound tissues on day 8 were homogenized using the appropriate buffer for each respective kit, and MDA and GSH levels were measured according to the manufacturer’s instructions provided with a GSH/GSSG Ratio Detection Assay Kit (Abcam, Cambridge, UK) and Lipid Peroxidation (MDA) Assay Kit (Abcam, Cambridge, UK).

### 2.15. Histological Analysis

The skin tissues collected from the sacrificed rats on day 8 post-wounding, using a 6 × 6 mm punch, were fixed in 10% neutral buffered formaldehyde. For subsequent analysis of the wound tissues, the samples were stained with Masson’s trichrome, hematoxylin and eosin (H&E), immunohistochemistry (IHC), and immunofluorescence (IF). IHC staining was performed using TUNEL and Ki67. IF staining was performed using CD31 (Abcam, Cambridge, UK). Histological observations were performed using a Nikon Eclipse Ti2 fluorescence live-cell imaging microscope (Nikon, Tokyo, Japan). The Masson’s trichrome, Ki67, TUNEL, and CD31-stained tissues were quantified using ImageJ software.

### 2.16. Statistical Analysis

The results are presented as the mean ± standard deviation (S.D.). Error bars mean the S.D. of the mean from each independent sample. The statistical significance of differences between groups was analyzed using post hoc tests with ANOVA. GraphPad Prism 8.0 (GraphPad Software, La Jolla, CA, USA) was used for all statistical analyses.

## 3. Results

### 3.1. Pinitol Promoted Proliferation and Wound Healing

**Figure 1:**
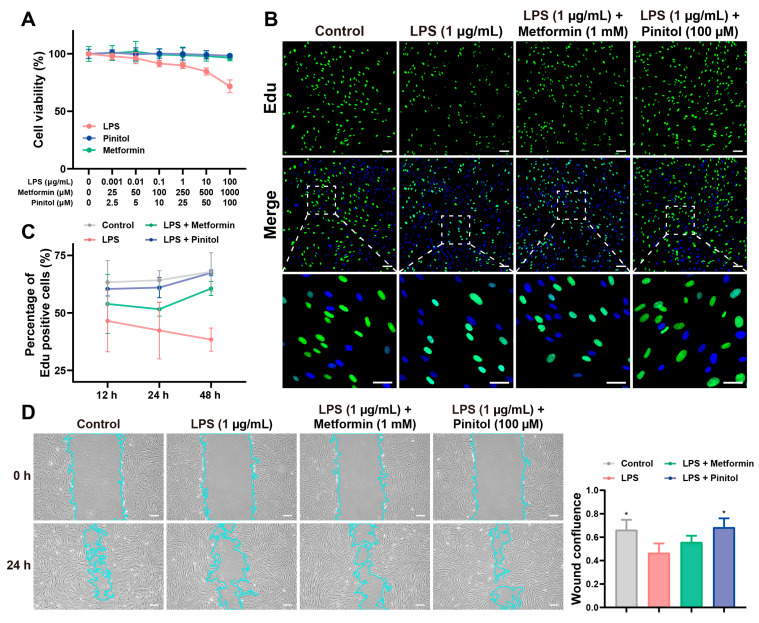
Pinitol promoted proliferation and wound healing in vitro. (**A**) The cytotoxic effect on HDFs was evaluated with different concentrations of pinitol, metformin, and LPS for 24 h (*n* = 6). (**B**,**C**) EdU staining (upper scale bar, 100 µm; lower scale bar, 50 µm) was used to detect cell proliferation after 48 h of culture. Cells were labeled with Edu to detect DNA synthesis (green). Nuclei were counterstained with DAPI to visualize total DNA (blue). Fluorescence immunoassay analysis measured cell proliferation at different time points: 12, 24, and 48 h (*n* = 3). (**D**) The wound-healing efficacy of pinitol was assessed in LPS-damaged HDFs. The boundary of cells (light blue) was marked. Statistical analysis of wound healing was conducted at 0 and 24 h (scale bar, 20 µm) (*n* = 3). Data are presented as mean values ± SD. * *p* < 0.05, compared with the LPS-treated group.

We assessed the cytotoxic effects of pinitol on HDFs. Metformin, a well-known antidiabetic drug [20,21], was used as a positive control, whereas LPS was used as a negative control to simulate a wound infection environment. Pinitol at concentrations from 2.5 to 100 μM, and metformin from 25 to 1000 μM, did not exhibit any cytotoxic effects on HDFs. As expected, LPS exhibited cytotoxicity (Figure 1A). Additionally, we performed an EdU assay to assess cell proliferation. The fluorescent images revealed that pinitol enhanced cell proliferation, highlighting its potential to promote cell growth (Figure 1B,C). A wound-healing assay was conducted to analyze the wound-healing potential of pinitol in LPS-damaged HDFs. By comparing the scratch width at 0 h and 24 h, we observed that the scratch closure in the pinitol-treated group was faster than in the LPS-treated group (Figure 1D). These findings suggest that pinitol promoted cell migration and proliferation of LPS-induced wound damage.

### 3.2. Pinitol Enhanced the Upregulation of Nrf2 to Mediate the Antioxidant Response

**Figure 2:**
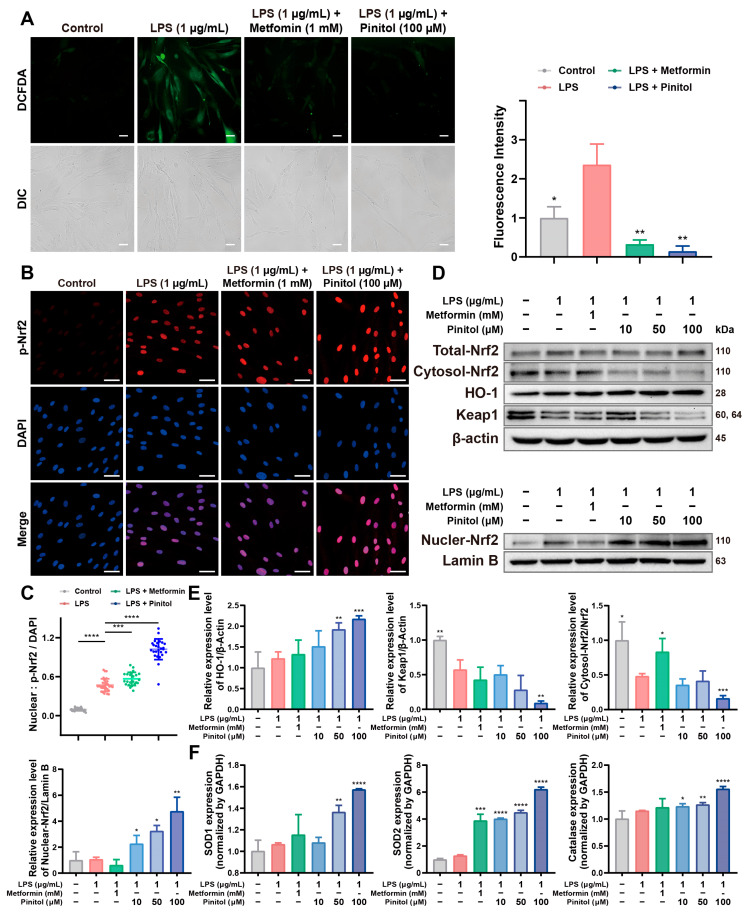
Pinitol mitigated intracellular ROS levels in LPS-damaged HDFs. (**A**) Intracellular ROS levels (green) were evaluated using the DCFDA assay (scale bars, 100 µm) (*n* = 3). (**B**,**C**) The localization of p-Nrf2 (red) was assessed through immunofluorescence analysis (scale bars, 50 µm) DAPI staining for cell nuclei (blue) was conducted. The merged image highlighted the colocalization of p-Nrf2 with nuclei (*n* = 3). Immunofluorescence analysis of p-Nrf2/DAPI values. (**D**,**E**) Western blot analysis confirmed that pinitol decreases cytosolic Nrf2 and Keap1 levels while increasing nuclear Nrf2 and HO-1 expression (*n* = 3). (**F**) The mRNA expression levels of antioxidant genes were measured (*n* = 3). Data are presented as mean values ± SD. * *p* < 0.05, ** *p* < 0.01, *** *p* < 0.001, **** *p* < 0.0001, compared with the LPS-treated group.

LPS stimulation causes oxidative stress, leading to harmful effects on cells [9,22]. The upregulation of Nrf2 mitigates oxidative stress and increases antioxidant enzyme levels [23,24]. Using the DCFDA assay, we measured intracellular ROS levels. As expected, we observed that LPS significantly increased ROS levels compared to the control, whereas pinitol treatment significantly reduced ROS levels (Figure 2A). To explore the molecular mechanism, we found that pinitol significantly increased the expression levels of Nrf2 (Figure 2B,C). These findings indicated that pinitol reduced the expression level of Keap1, thereby enhancing Nrf2 translocation to the nucleus. We further demonstrated that this activation of Nrf2 in the nucleus increased the expression level of HO-1, promoting the antioxidant effects of pinitol (Figure 2D,E). In addition, we observed that pinitol enhanced the upregulation of antioxidant proteins, such as SOD1, SOD2, and catalase, through Nrf2 activation (Figure 2F). These results suggest that pinitol activated the Keap1/Nrf2/HO-1 signaling pathway and mitigated LPS-induced oxidative stress.

### 3.3. Pinitol Downregulated the IκBα/NF-κB Signaling to Reduce LPS-Induced Inflammation

**Figure 3:**
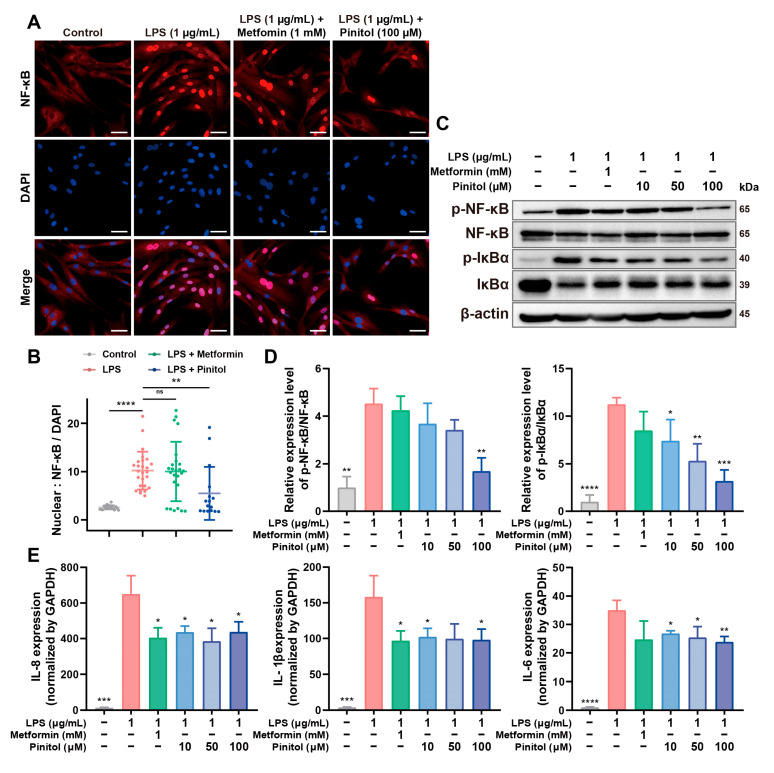
Pinitol inhibited inflammatory response in LPS-damaged HDFs. (**A**,**B**) The expression level of NF-κB (red) was assessed through immunofluorescence analysis (scale bar, 50 µm). DAPI stating for cell nuclei (blue) was performed. The merged image highlighted the colocalization of NF-κB with nuclei (*n* = 3). Immunofluorescence analysis of NF-κB/DAPI values. (**C**,**D**) Western blot analysis confirmed that pinitol reduces the activation of NF-κB, and IκBα (*n* = 3). (**E**) The mRNA expression levels of proinflammatory cytokine genes were measured (*n* = 3). Data are presented as mean values ± SD. * *p* < 0.05, ** *p* < 0.01, *** *p* < 0.001, **** *p* < 0.0001, compared with the LPS-treated group.

LPS exposure activates the IκBα/NF-κB signaling pathway, triggering inflammatory responses, oxidative stress, and the production of pro-inflammatory cytokines, including IL-6, IL-8, and IL-1β [25,26]. We evaluated NF-κB activation by observing nuclear translocation using immunofluorescence. LPS treatment increased nuclear fluorescence compared to the control, whereas pinitol significantly inhibited NF-κB activation (Figure 3A,B), suggesting that pinitol inhibited the translocation of NF-κB to the nucleus. 

Western blot analysis confirmed that pinitol treatment reduced NF-κB activation and also decreased IκBα levels (Figure 3C,D), indicating that pinitol inhibited the IκBα/NF-κB pathway. Furthermore, real-time qRT-PCR analysis demonstrated that LPS increased mRNA levels of pro-inflammatory cytokines (IL-6, IL-8, and IL-1B), whereas pinitol significantly reduced these levels (Figure 3E), indicating that pinitol alleviated LPS-induced inflammatory responses by inhibiting the IκBα/NF-κB signaling pathway.

### 3.4. Pinitol Improved LPS-Induced Mitochondrial Dysfunction

Oxidative stress induced by LPS leads to mitochondrial dysfunction and triggers cell death [27,28]. We investigated the protective effects of pinitol in mitigating mitochondrial damage under these conditions. 

**Figure 4:**
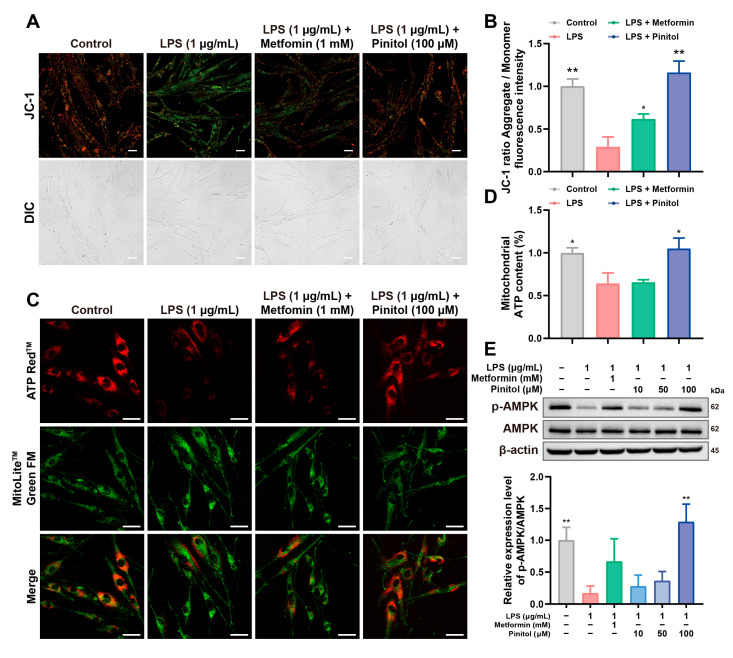
Pinitol improved mitochondrial dysfunction in LPS-damaged HDFs. (**A**,**B**) Mitochondrial membrane potential was evaluated using the JC-1 assay (scale bar, 100 µm) (*n* = 3). The fluorescence intensities of J-monomers (green) and J-aggregates (red) were captured by a fluorescence microscope and analyzed using ImageJ™ software (**C**,**D**) Mitochondrial ATP production capacity was assessed using the ATP assay (scale bar, 50 µm) (*n* = 3). Fluorescence microscopy images revealed red fluorescence representing ATP levels and green fluorescence marking mitochondria. The merged image highlighted the colocalization of ATP within mitochondria, indicating the cellular energy status and mitochondrial integrity (**E**) Western blot analysis exhibited that pinitol increased the activation of AMPK (*n* = 3). Data are presented as mean values ± SD. * *p* < 0.05, ** *p* < 0.01, compared with the LPS-treated group.

Using the JC-1 assay, we observed JC-1 aggregation as red fluorescence in HDFs, indicating a healthy mitochondrial membrane potential (ΔΨm). In contrast, LPS treatment increased monomeric JC-1, indicating a loss of ΔΨm [29]. However, pinitol treatment improved ΔΨm, suggesting a protective effect on mitochondrial function in LPS-damaged HDFs (Figure 4A,B). Furthermore, ATP levels were found to decrease in LPS-induced HDFs. In contrast, pinitol treatment restored ATP levels to those observed in normal HDFs (Figure 4C,D). AMPK is a critical enzyme that connects energy sensing with metabolic regulation. Therefore, dysfunction of AMPK negatively impacts metabolism, mitochondrial function, and the cell life cycle [30,31,32]. Western blot analysis showed that pinitol treatment enhanced the expression level of AMPK (Figure 4E), which correlated with the improvement in mitochondrial function. In summary, pinitol effectively mitigated mitochondrial dysfunction induced by LPS.

### 3.5. Pinitol Downregulated the MAPK Pathway, Reduced MMP Levels, and Protected Collagen

LPS treatment significantly increases the activity of MAPKs, causing signaling pathways in inflammation and cellular stress responses [33,34]. Furthermore, the upregulation of MAPKs enhances MMP expression, leading to collagen degradation and suppressing TIMP expression, thereby disrupting the balance of extracellular matrix homeostasis [35,36]. 

**Figure 5:**
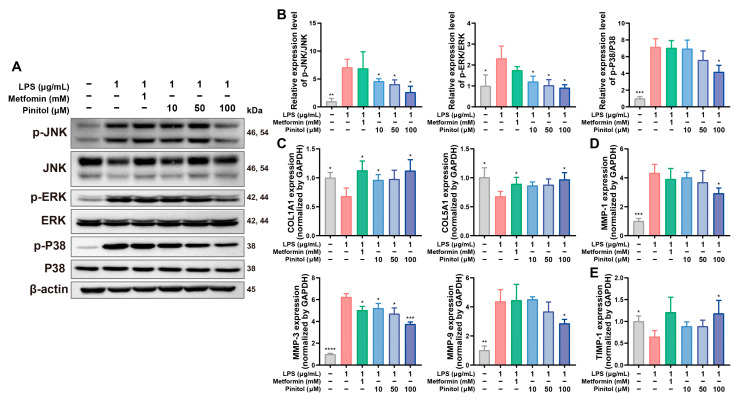
Pinitol inhibited MAPK activation and increased collagen production in LPS-damaged HDFs. (**A**,**B**) Western blot analysis confirmed that pinitol inhibited the activation of MAPK family proteins (*n* = 3). (**C**–**E**) The mRNA expression levels of several collagen, MMP, and TIMP1 genes were measured (*n* = 3). Data are presented as mean values ± SD. * *p* < 0.05, ** *p* < 0.01, *** *p* < 0.001, **** *p* < 0.0001, compared with the LPS-treated group.

Western blot analysis confirmed that LPS treatment significantly increased MAPK activation, whereas pinitol treatment inhibited MAPK activation protein levels (Figure 5A,B). Consequently, real-time qRT-PCR analysis revealed an increase in the gene expression levels of collagen and TIMP1, and a decrease in MMP gene levels (Figure 5C–E). These findings suggest that pinitol suppressed the MAPK activation induced by LPS, thereby reducing MMP expression and promoting the expression of TIMP1 and collagen.

### 3.6. Pinitol Accelerated Wound Healing in Diabetic Foot Ulcers

**Figure 6:**
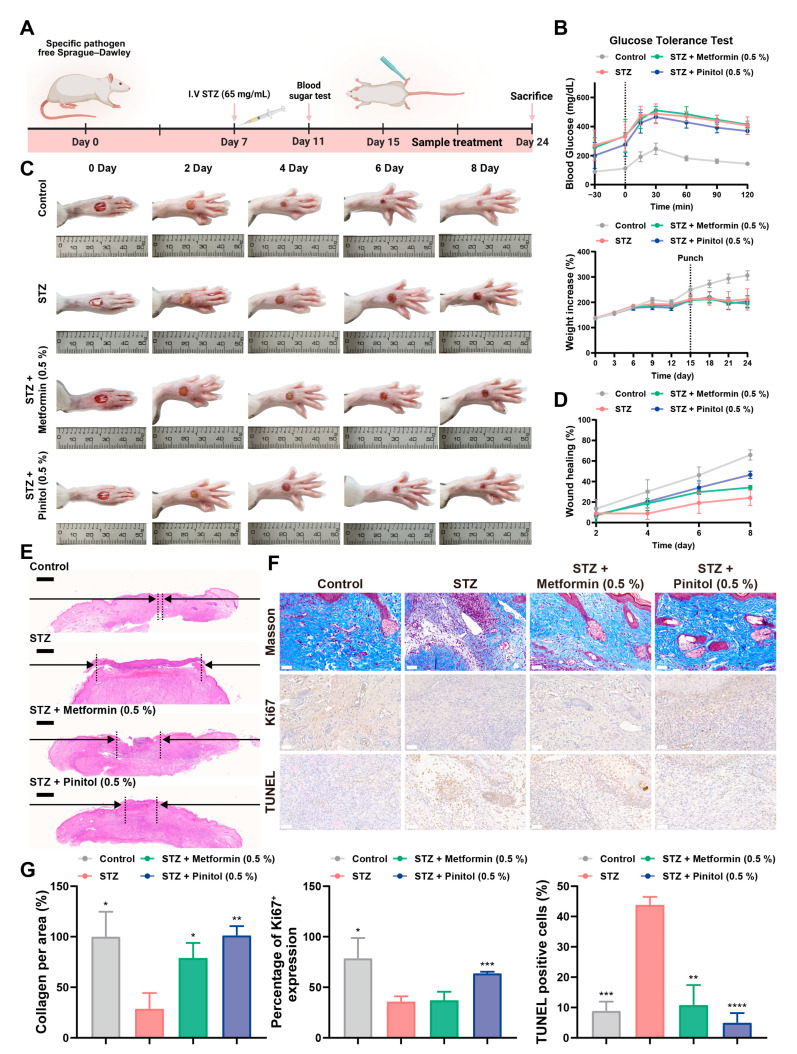
Pinitol promotes wound healing in STZ-induced diabetic foot ulcers (**A**) Schematic representation of the wound-healing experiment in a diabetic foot ulcers rat model. (**B**) Glucose tolerance test results for each group on Day 11 and body weight measurements for each group from the start to the end of the experiment (*n* = 6). The dashed line in the upper figures represented the first glucose administration, whereas the dashed line in the lower figure indicated punch initiation (**C**,**D**) Representative images of wounds for each group were captured on days 0, 2, 4, 6, and 8; results are presented as wound-healing rates (*n* = 6). (**E**) H&E staining (scale bar, 300 μm). The space between the arrows indicated the size of a wound closure. (**F**,**G**) Masson’s trichrome staining for highlighting collagen fibers (blue) was conducted (scale bar, 50 μm). Immunohistochemistry for Ki67, and TUNEL analysis (scale bar, 50 μm) was performed on tissue sections with the indicated antibodies; quantitative measurements of collagen index, Ki67, and TUNEL were conducted (*n* = 3). Data are presented as mean values ± SD. * *p* < 0.05, ** *p* < 0.01, *** *p* < 0.001, **** *p* < 0.0001, compared with the STZ-induced diabetes group.

The effect of pinitol on wound healing was evaluated using SD rats in an STZ-induced diabetic foot ulcer model (Figure 6A,B) [19,37]. Rats were treated with 0.5% pinitol applied topically to the wound area for 8 days, and wound-healing progression was assessed every 2 days from day 0 to day 8 (Figure 6C). By day 8. the wound-healing percentages were as follows: control (65.86 ± 5.00), STZ (24.11 ± 7.54), metformin (34.13 ± 2.09), and pinitol (46.54 ± 3.53). Compared to the STZ-treated group, the pinitol-treated group exhibited significantly improved wound healing (Figure 6D). Furthermore, H&E staining analysis revealed that the pinitol-treated group showed reduced epithelial wound gaps, indicating accelerated wound healing (Figure 6E). Masson’s trichrome staining confirmed increased collagen deposition in the pinitol-treated group compared to the STZ-treated group. Ki67 and TUNEL assays also demonstrated that pinitol enhanced cell proliferation and reduced apoptosis (Figure 6F,G). These findings suggest that pinitol promoted wound healing and inhibited cell death in diabetic foot ulcers.

### 3.7. Pinitol Upregulated Nrf2 to Reduce Oxidative Stress and Inflammation, Enhancing Diabetic Foot Ulcers

**Figure 7:**
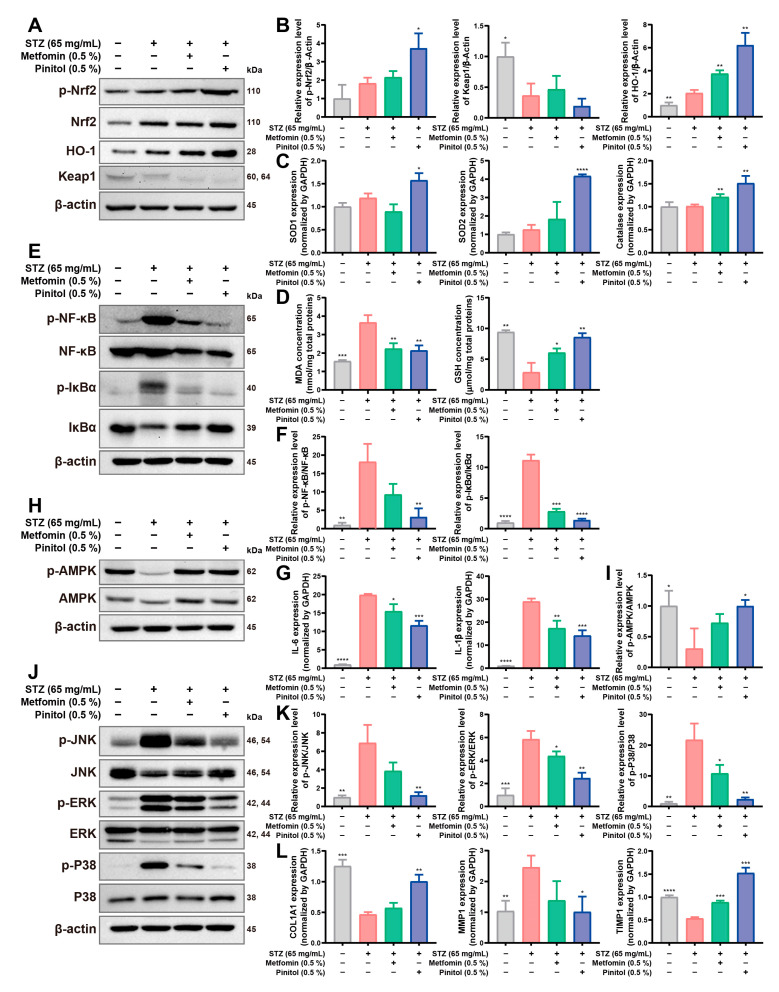
Pinitol promoted healing through the activation of Nrf2 in STZ-induced diabetic foot ulcers. (**A**,**B**) Western blot analysis exhibited a decrease in Keap1 and an increase in the expression levels of Nrf2 and HO-1 in tissues of foot wounds (*n* = 3). (**C**) The mRNA expression levels of antioxidant genes in foot wound tissue were measured (*n* = 3). (**D**) MDA and GSH levels were measured in foot wound tissue (*n* = 3). (**E**,**F**) Western blot analysis revealed that pinitol reduces the activation of NF-κB and IκBα in tissues of foot wounds. (**G**) The mRNA expression levels of proinflammatory cytokine genes in foot wound tissue were measured. (**H**,**I**) Western blot analysis exhibited that pinitol increased the activation of AMPK in tissues of foot wounds. (**J**,**K**) Western blot analysis revealed that pinitol inhibited the activation of MAPK family proteins (*n* = 3). (**L**) The mRNA expression levels of collagen, MMP, and TIMP1 genes were measured (*n* = 3). Data are presented as mean values ± SD. * *p* < 0.05, ** *p* < 0.01, *** *p* < 0.001, **** *p* < 0.0001, compared with the STZ-induced diabetes group.

We investigated the molecular mechanisms by which pinitol improved wound healing in diabetic foot ulcers. On day 8, skin tissue from the foot wounds of diabetic rats was collected using a 6 × 6 mm punch biopsy, followed by Western Blot and real-time qRT-PCR analyses. In diabetic wound tissues, an increase in Nrf2 expression was observed in the pinitol-treated group compared to the untreated group. Activation of HO-1, SOD1, SOD2, and catalase (Figure 7A–C) led to an increase in GSH levels and a decrease in MDA levels (Figure 7D), indicating that pinitol-mediated upregulation of Nrf2 alleviated oxidative stress. Inflammatory responses in diabetic patients can significantly hinder wound healing [38,39]. Pinitol suppressed the IκBα/NF-κB signaling pathway (Figure 7E,F), thereby reducing the expression levels of IL-6 and IL-1β (Figure 7G). Oxidative stress in diabetics can impair AMPK function, contributing to diabetic complications [27,30]. AMPK function was restored in the pinitol-treated group, enhancing mitochondrial function and energy homeostasis (Figure 7H,I). Oxidative stress and inflammation in diabetics can increase MAPK activation, leading to reduced activities of COL1 and TIMP1 [40,41]. Pinitol treatment inhibited MAPK activation (Figure 7J,K), thereby enhancing the expression levels of COL1A1 and TIMP1, while downregulating the expression level of MMP1 (Figure 7L). These findings suggest that pinitol improved wound recovery in diabetic foot ulcers by alleviating oxidative stress, reducing inflammation, and restoring metabolic function through the upregulation of Nrf2.

### 3.8. Pinitol Restored Impaired Angiogenesis in Diabetic Foot Ulcers

**Figure 8:**
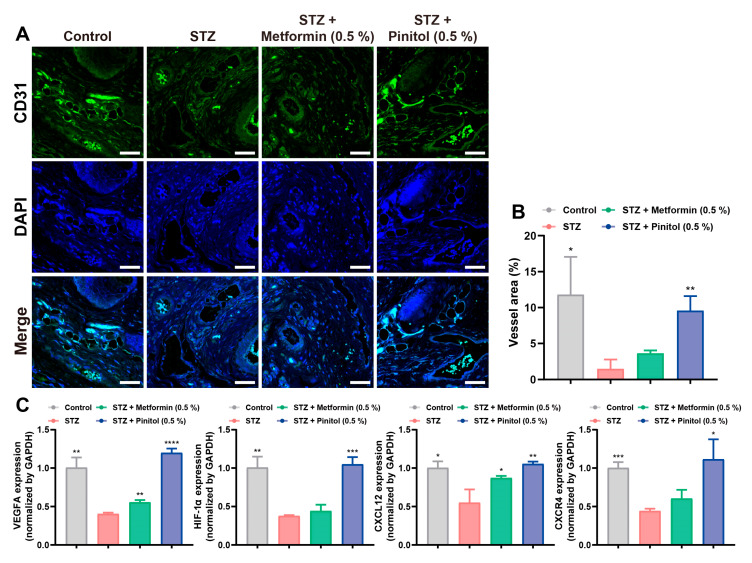
Pinitol promoted angiogenesis in STZ-induced diabetic foot ulcers. (**A**,**B**) Immunofluorescence analysis (scale bar = 50 μm) was performed on foot wound tissue with the indicated antibodies; statistical analysis of CD31 expression (green). DAPI staining for cell nuclei (blue) in foot wound tissue was also conducted (*n* = 3). (**C**) The mRNA expression levels of angiogenesis genes were measured. Data are presented as mean values ± SD. * *p* < 0.05, ** *p* < 0.01, *** *p* < 0.001, **** *p* < 0.0001, compared with the STZ-induced diabetes group.

Angiogenesis plays a critical role in wound healing. In diabetes, impaired angiogenesis significantly delays the wound-healing process [42,43,44]. The expression level of the vascular endothelial cell marker CD31 was lower in diabetic wound tissues, indicating a reduced number of endothelial cells compared to normal wound tissue. Pinitol treatment increased CD31 expression, thereby enhancing the number of vascular endothelial cells (Figure 8A,B). Furthermore, pinitol upregulated the expression levels of angiogenesis-related genes, including VEGFA, HIF-1A, CXCL12, and CXCR4, thereby promoting angiogenesis (Figure 8C). These findings suggest that pinitol improved impaired angiogenesis in diabetes, contributing to enhanced wound healing.

## 4. Discussion

Diabetic foot ulcers are a severe complication for diabetic patients, often leading to limb amputation and, in extreme cases, death [45]. Treating diabetic foot ulcers is particularly challenging due to the complex healing mechanisms involved, despite the availability of various therapeutic options such as immunotherapy, stem cell therapy, growth factor therapies, traditional ointments, and skin substitutes. Effective clinical management and assessment of diabetic foot ulcers remain persistent challenges for healthcare providers [46,47].

In an STZ-induced rat model of diabetic foot ulcers, the pinitol-treated group demonstrated significantly enhanced wound healing compared to the untreated group. This improvement was attributed to the ability of pinitol to reduce oxidative stress, suppress inflammatory responses, and inhibit cell apoptosis at the wound site. Furthermore, pinitol promoted cell migration, proliferation, and overall wound healing.

Nrf2 plays a crucial role in wound healing [48,49]. A deficiency in Nrf2 delays wound healing and leads to prolonged overexpression of inflammatory cytokines, ultimately reducing collagen production. Moreover, patients with Nrf2 gene mutations are more susceptible to diabetic complications, including peripheral neuropathy, nephropathy, retinopathy, foot ulcers, and microangiopathy [50]. Activation of Nrf2 modulates diverse responses to oxidative stress and plays an essential role in cell migration, proliferation, apoptosis, and differentiation [51,52]. Additionally, upregulation of Nrf2 has shown therapeutic effects in diabetic nephropathy mouse models [51,53]. Therefore, Nrf2 is a promising therapeutic target for treating diabetic foot ulcers.

In this study, we cultured HDFs in 25 mM glucose DMEM medium and treated them with LPS to induce a wound infection model. We then assessed whether pinitol could promote recovery by activating Nrf2 in this infected model. Pinitol increased the nuclear translocation of Nrf2 and significantly upregulated antioxidant enzymes, such as HO-1, SOD1, SOD2, and catalase, thereby reducing oxidative stress. Moreover, the upregulation of Nrf2 by pinitol inhibited the IκBα/NF-κB signaling pathway, resulting in a reduction in inflammatory cytokines, including IL-6, IL-1β, and IL-8. Furthermore, pinitol improved energy metabolism and mitochondrial dysfunction while suppressing MAPK activation, thereby preventing cell apoptosis. This inhibition also decreased the expression levels of MMP1, MMP3, and MMP9, ultimately enhancing collagen production. These findings indicate that pinitol-mediated upregulation of Nrf2 promoted wound-healing effects in wound infection models.

In the STZ-induced diabetic rat model of foot ulcers, pinitol treatment significantly reduced wound size compared to untreated controls. Pinitol enhanced cell migration and proliferation, inhibited cell apoptosis, increased collagen production, and promoted angiogenesis in diabetic foot ulcers. Additionally, it suppressed inflammatory responses and enhanced antioxidant enzyme activity, leading to improved wound healing. These effects were closely associated with the Nrf2 induction.

## 5. Conclusions

**Figure 9:**
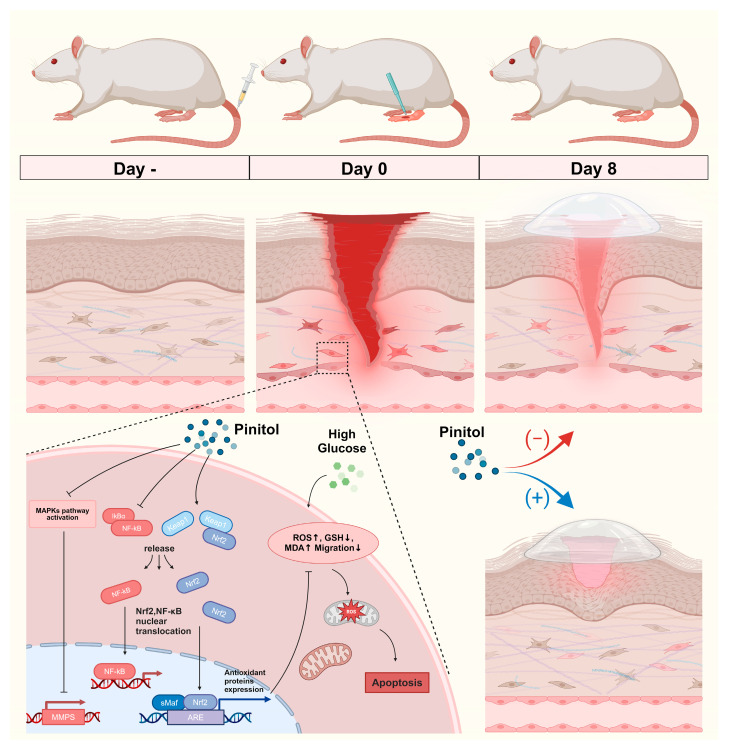
Schematic representation of pinitol-mediated treatment for diabetic foot ulcers.

In conclusion, we demonstrated that pinitol enhanced wound healing in diabetic foot ulcers by activating Nrf2, which increased antioxidant enzyme levels and reduced oxidative stress. Additionally, pinitol suppressed inflammatory responses by downregulating the NF-κB/IκBα pathway and improving cell migration and proliferation (Figure 9). These findings suggest that pinitol holds promise as a potential therapeutic agent for treating diabetic foot ulcers, offering a complementary approach to current therapies and potentially reducing the burden of chronic wound-related complications.

## Data Availability

The data from this study are available upon demand from the corresponding author.

## Abbreviations

HDFs, human dermal fibroblast; FBS, fetal bovine serum; DMEM, high-glucose Dulbecco’s modified Eagle’s medium; LPS, lipopolysaccharide; UV, ultraviolet; DPBS, Dulbecco’s phosphate buffered saline; DCFDA, 2′,7′-dichlorodihydrofluorescein diacetate; ROS, reactive oxygen species; FITC, fluorescein isothiocyanate; RITC, rhodamine B isothiocyanate; JC-1, 5,5′,6,6′-tetrachloro-1,1′,3,3′-tetraethylbenzimidazolocarbo-cyanine iodide; BCA, IHC, immunohistochemistry; IF, immunofluorescence; bicinchoninic acid; SDS, sodium dodecyl sulfate; ERK, extracellular signal-regulated kinase; JNK, c-Jun N-terminal kinase; NF-κb, nuclear factor kappa B; IκBα, NF-kappa-B inhibitor alpha; HO-1, heme oxygenase 1; Keap1, Kelch-like ECH-associated protein 1; Nrf2, nuclear factor erythroid 2-related factor 2; STZ, streptozotocin; MDA, malondialdehyde; GSH, glutathione; SOD1, superoxide dismutase type 1; SOD, superoxide dismutase type 2; IL-1β, interleukin-1 beta IL-6, interleukin-6; IL-8, interleukin-8; AMPK, 5′ adenosine monophosphate activated protein kinase; COL1A, collagen type I alpha 1; MMP1, matrix metallopeptidase 1; TIMP1, TIMP metallopeptidase inhibitor 1; TUNEL, terminal deoxynucleotidyl transferase dUTP nick end labeling; CD31, Cluster of Differentiation 31; VEGFA, vascular endothelial growth factor A; HIF-1α, hypoxia-inducible factor 1 alpha; CXCL12, C-X-C motif chemokine ligand 12; CXCR4, C-X-C chemokine receptor 4.
